# Differences in myoelectric activity of the lumbar muscles between recurrent and chronic low back pain: a cross-sectional study

**DOI:** 10.1186/s12891-021-04623-9

**Published:** 2021-09-03

**Authors:** Balasch-Bernat Mercè, Willems Tine, Danneels Lieven, Meeus Mira, Goubert Dorien

**Affiliations:** 1 Department of Physiotherapy, University of Valencia, Valencia, Spain; 2 Physiotherapy in Motion, MultiSpeciality Research Group (PTinMOTION), University of Valencia, Valencia, Spain; 3 Department of Rehabilitation Sciences, Faculty of Medicine and Health Sciences, Ghent University, Ghent, Belgium; 4 Department of Rehabilitation Sciences, Campus Heymans (UZ) 3 B3, Corneel Heymanslaan 10, 9000 Ghent, Belgium; 5 Pain in Motion Research Group, Valencia, Spain; 6 Department of Rehabilitation Sciences and Physiotherapy, Faculty of Medicine and Health Sciences, University of Antwerp, Antwerp, Belgium

**Keywords:** low back pain, lumbar muscles, recurrence, chronic pain, muscle activity

## Abstract

**Background:**

Altered lower back muscle activity is proposed as a contributing factor to the reoccurrence and chronicity of low back pain (LBP). This study compared lumbar muscle activity during trunk extension in patients with continuous chronic LBP (CLBP), non-continuous CLBP, recurrent LBP (RLBP) and healthy subjects.

**Methods:**

In 75 subjects (16 continuous CLBP, 15 non-continuous CLBP, 23 RLBP, 21 healthy controls), surface electromyographic (EMG) activity of the lumbar erector spinae (ES), multifidus (MF), latissimus dorsi (LD) and gluteus maximus (GM) was recorded during the concentric, holding and eccentric phase of a modified Biering Sorenson exercise.

**Results:**

Continuous CLBP patients showed higher EMG activity in the ES and MF muscles compared to healthy controls in the concentric (*p* = 0.011; *p* = 0.009 respectively) and the holding phase (*p* = 0.015; *p* = 0.013). Higher EMG activity was observed in continuous CLBP compared to RLBP in the ES and MF muscles in the holding phase (*p* = 0.035; *p* = 0.037), and in the MF in the concentric phase (*p* = 0.046), but not in the ES (*p* = 0.062). No differences in muscle activity were established in either the concentric, holding, and eccentric phase for the LD and GM muscles. No differences were found between non-continuous CLBP and the other groups.

**Conclusions:**

An enhanced muscle activity of the lumbar muscles during the concentric and holding phase was observed during trunk extension in patients with continuous CLBP compared to patients with RLBP and healthy subjects. No differences between groups are present in the GM and LD muscles during concentric and holding phases and for any muscle in the eccentric phase.

## Background

Recurrence is common in people with low back pain (LBP) [1], but it remains unclear why some patients recover after every LBP episode while others do not.

Altered back muscle activity has been proposed as a factor contributing to LBP. Several lumbar muscles such as erector spinae (ES), multifidus (MF), latissimus dorsi (LD) and gluteus maximus (GM) play an important role in the stabilization and dynamic control of the lumbar spine [2, 3].

Numerous studies have reported changes in the back muscle activity patterns in both patients with recurrent and chronic LBP. Enhanced activity of the superficial back muscles has been reported in people with chronic LBP (CLBP) compared to healthy controls [4–6] as a compensatory strategy to increase spinal stability, leading to fatigability of the spinal muscles [7, 8]. In recurrent LBP (RLBP) in remission previous research observed reduced muscle activity compared to healthy subjects [9, 10], whereas others found the opposite [11].

Currently, differences in lumbar muscle activity between patients with recurrent and chronic LBP are still unclear. It can be hypothesised that different mechanisms occur in both LBP populations. A recent study established enhanced metabolic activity in CLBP patients compared to RLBP patients [12]. It is therefore possible that also differences in electrical activity occur between RLBP and CLBP patients. However, to date, there are no studies investigating the difference in electrical activity patterns between RLBP and CLBP.

Furthermore, heterogeneity amongst CLBP patients is known. Some authors suggest that the CLBP group consists of several subgroups, each marked by different characteristics [13] and differences in fat infiltration in the lumbar muscles has been established between LBP patients with different level of recurrence [12]. It is therefore reasonable that muscle activity might also differ between CLBP subgroups and that lumbar muscle activity depends on the level of recurrence of LBP.

Hence, this study aims to compare lumbar muscle activity in the ES, MF, LD and GM during trunk extension in patients with different LBP persistence levels and healthy subjects.

## Methods

We hypothesized that LBP might represent itself as a spectrum in which electrical muscle activity is normal in healthy persons and alterations in muscle activity is directly proportional to the continuation of pain complaints.

### Participants

This comparative cross-sectional study was part of a larger study of which results of other endpoints have been published elsewhere [12, 14]. Subjects with non-specific LBP and healthy subjects were recruited through advertising in social media and healthcare settings such as different hospitals in Ghent and private practices. All in- and exclusion criteria are presented in Table 1 and they were defined in a previous study [12]. An exhaustive anamnesis was performed by the investigators to verify eligibility to all in-and exclusion criteria, including pain and disability at the moment of testing, start of the symptoms, number and frequency of the episodes in the past year and in the last week, duration and pain intensity during pain flares and duration and degree of alleviation of the symptoms and disability during the pain free episodes. These data was used by the investigators to divide participants in the different study groups (healthy subjects, RLBP, non-continuous CLBP and continuous CLBP). On the assessment day, all participants were asked to refrain from alcohol, nicotine, caffeine and all medication. Subjects were also instructed not to perform exhausting physical activities the day before. All participants signed informed consent prior to participation.

**Table 1:** In- and exclusion criteria

Group	Specific inclusion criteria	General inclusion criteria	General exclusion criteria
**Healthy subjects**	- no history of LBP of that kind that a doctor or physiotherapist was ever consulted - fully pain-free at the moment of testing and 24 h before reflected by 0/10 on the NRS scale and 0 on the Rolland Morris disability questionnaire	- males and females - 18–65 years old - ≥ 1 years post-natal	- use of antidepressants or analgesics (except for NSAID’s or paracetamol), taken two weeks prior to the testing - neurological, respiratory, circulatory or severe orthopaedic diseases - back surgery - pregnancy - motor control training for LBP
**RLBP**	- non-specific RLBP in remission - ≥ 6 months - a frequency of ≥ 2 episodes in the past year - a pain flare of ≥ 24 h, characterized by an increase of ≥ 2 on a NRS scale and/or ≥ 5 on the Rolland Morris Disability Questionnaire - followed by a pain free episode of ≥ 1 month, characterized by a 0/10 on an NRS scale and/or < 2 on the Rolland Morris disability questionnaire - applicate for medical help concerning low back complaints		
**Non-continuous CLBP**	- non-specific CLBP - ≥ 3 months - 3 to 4 pain days a week		
**Continuous CLBP**	- non-specific CLBP - ≥ 3 months - 7 pain days a week		

### Exercise protocol

**Fig. 1:**
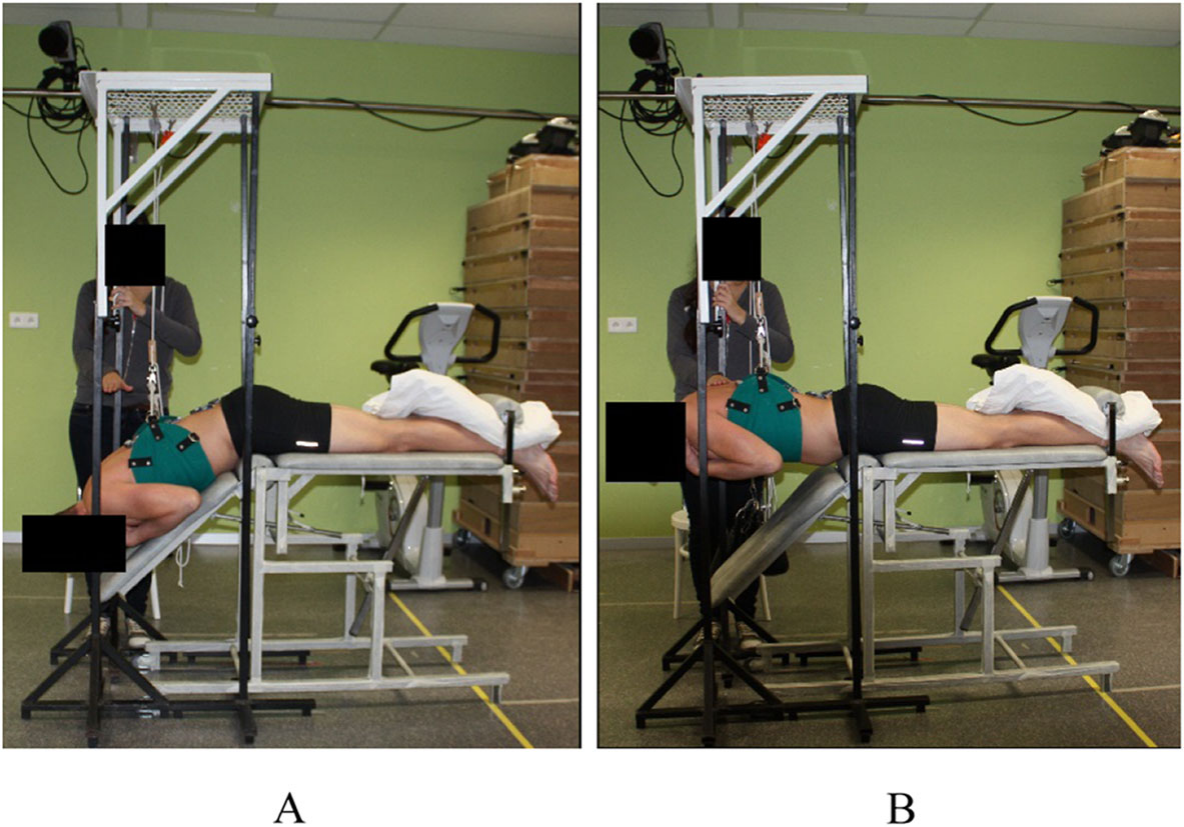
Static-dynamic extension exercise at 40 % of 1RM. **A**, start position, this is the beginning of the concentric phase (and the end of the eccentric phase); **B**, the holding phase. Subjects were in prone, with a trunk flexion of 45°. The legs were strapped to the table and the hands of the subject were placed on the ipsilateral shoulders. They raised the upper body in 2 s, holded it for 5 s at the horizontal position and lowered the upper body to the start position in 2 s. The performance was adjusted by use of tactile feedback of the assessor and a metronome was used to ensure appropriate timing (60 beats/minute). Exercise volume and load were set at 10 repetitions of 40 % of the subjects’ personal one repetitions maximum (1RM) (Fig. 1). The 1RM was indirectly determined from the maximum amount of trunk extensions performed with the subjects own upper body weight, which was assessed at least 2 h before the EMG measurement in order to avoid muscle fatigue. The Holten diagram was used to calculate the exercise weight corresponding to the personal 40 % of 1RM. The exercise weight was lower compared to the subject’s trunk weight. Therefore a load-pulley system assisted in performing the trunk extensions by use of assisted weight [22]

In this study, participants had to perform 10 repetitions of the modified Biering-Sorenson test on a variable angle table (Fig. 1), which was previously described [12].

### EMG measurement

**Fig. 2:**
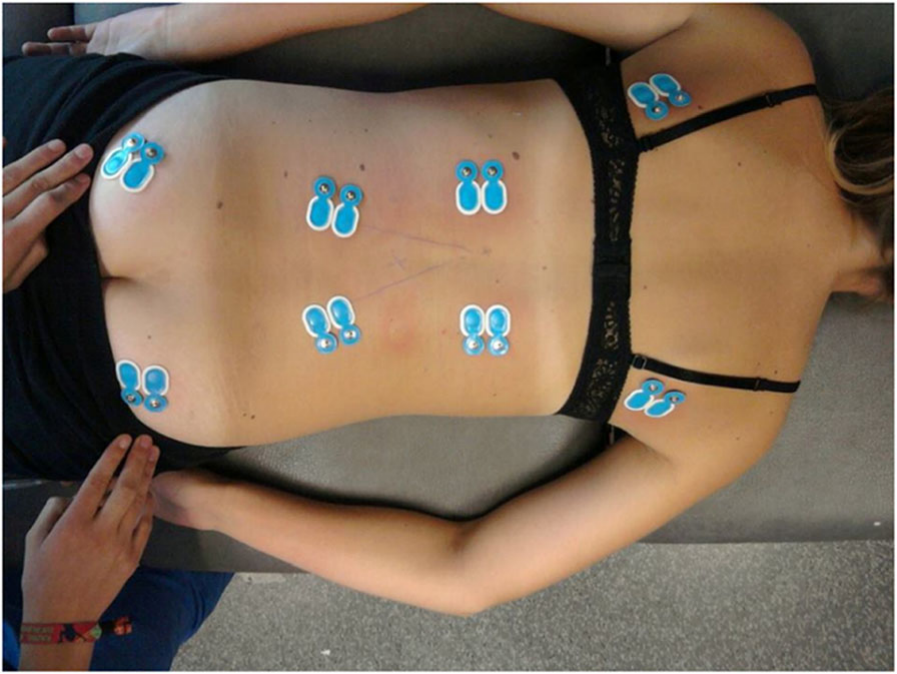
Placement of the surface electrodes. The electrodes were placed on the LD (1 cm lateral and inferior of the inferior scapular angle), ES (at level L1), MF (at level L5 on the line drawn from the posterior inferior iliac spine to the intervertebral space between level L1-L2) and GM (at the middle point between the posterior inferior iliac spine and the ischial tuberosity), with the patient in prone lying position. Placements of electrodes were decided based on the SENIAM recommendations (www.seniam.org) and expert opinions. Before admission of the surface electrodes (Ambu A/S, Denmark), skin preparation occurred by shaving, scrubbing and cleansing with alcohol. After electrode placement, 8 amplifiers were attached to the skin in the proximity of the measuring site. These amplifiers captured and amplified the electric signal prior to forwarding it to the desk receiver

Muscle activity of the LD, ES, MF and GM was bilaterally recorded by surface EMG during the modified Biering Sorenson exercise, using a wireless Noraxon Direct transmission service system (Noraxon U.S.A. Inc., Arizona) (Fig. 2). The EMG activity of the ES and MF were measured at levels L1 and L5, respectively.

Before the execution of the extension exercises, 3 maximal voluntary contraction (MVC) trials of 4 s duration, with 30 s of rest between each trial, were captured per muscle group. This procedure was conducted according to the Noraxon guidelines. All tests were performed with the patient in prone position. MVC’s for LD and GM were measured at both right and left side [15]. To obtain the MVC of the LD, participants were lying with their arms in internal rotation while maximal resistance was applied proximal of the elbows against extension of the arm. To measure the MVC of the GM, the subjects were placed with 90º flexion of the knee of the tested side and the opposite leg was strapped to the table. Then, hip extension was maximally resisted proximal to the knee. During MVC measurement of the ES and MF, the subjects’ legs were strapped to the table at the middle of the calves. Subjects placed the back of their hands on their forehead while maximal resistance for trunk extension was given on the inferior angels of both scapulae [16, 17].

### EMG analysis

The raw EMG signals were submitted to electrocardiography and high pass filtering (20 Hz), rectification and smoothing using a root mean square algorithm with a 100 ms time constant. The highest activity of the MVC’s performed during 3 s was analysed. Two markers were placed for each contraction type of the exercise (one at the beginning and one at the end of the concentric, isometric and eccentric contraction) and the interval between each pair of markers was considered for further analysis. To evaluate the intensity of the muscle activity during each phase of the exercise, Root Mean Square calculations and post hoc assembly of the average activation patterns were calculated. The average activity signals were normalized to MVC voltage. All EMG data-processing was performed using the MR3.10 software (Noraxon U.S.A. Inc., Arizona) [15, 18].

### Pain measures, perceived exertion and disability

All subjects were asked to indicate the site of the symptoms (right, left or bilateral) as well as to rate their current pain intensity before, during and immediately after exercise performance by means of a Numerical Rating Scale (“0”= no pain, “10”=the worst pain imaginable). In addition, before the exercise, participants were asked to rate their expected pain from the exercise. After the exercise the rate of perceived exertion (RPE) on a BORG scale (ranging 6–20) was also registered. Disability on the day of assessment was assessed by the Roland-Morris disability questionnaire, representing the daily physical activities and functions affected by LBP complaints [19]. Pain scales and disability questionnaires were used for either RLBP, non-continuous CLBP and continuous CLBP participants.

### Statistical analysis

Statistics were performed in SPSS (IBM SPSS statistics, version 24.0). Normality of all demographical and movement parameters was checked visually and through the Shapiro-Wilk test. Since the data were not normally distributed, differences between groups for demographical parameters, pain measurements, perceived exertion rate, disability, and assisted weight were analysed by the Kruskall-Wallis test, whereas a Mann-Whitney U test was used for post-hoc pairwise comparison in case of significant differences between groups. The Pearson Chi-Square was used to assess differences between groups for sex distribution and symptom site. To counter multiple testing, significance was set at α < 0.01.

A mixed model analysis was performed to compare EMG activity of the different muscles between subjects. Model selection and validation was based on statistical tests for parameter estimates, comparison of Akaike’s Information Criterion values and inspection of residual plots. These mixed models account for correlated measures by including a random intercept for “subject” and “side” (left and right) and were adjusted for “group” (RLBP, non-continuous CLBP, continuous CLBP and healthy subjects) and “phase” (concentric, holding and eccentric phase). Interaction effects between group and phase were investigated. Furthermore, age as a covariate appeared not influential on the outcome for EMG. No other relevant covariates were taken into account. Parameter estimation was performed by restricted maximum likelihood and estimated means were corrected by Bonferroni to adjust for multiple comparison. Significance was set at α < 0.05.

## Results

### Demographics

Seventy-five subjects participated in this study of which 16 had continuous CLBP, 15 non-continuous CLBP, 23 RLBP and 21 healthy controls. All patient characteristics can be found in Table 2. There was a significant difference between the groups for age (*p* = 0.011). Post-hoc comparisons showed that patients with continuous CLBP were significantly older compared to patients with RLBP (*p* = 0.002) while there was no difference between the other groups. No differences between any groups were noted for BMI, sex and assisted weight.

**Table 2:** Descriptives on demographic variables, pain measurements, rate of perceived exertion and amount of assisted weight during the exercise. All values, expect sex and symptom site, are expressed by median and range. (HC = healthy controls; RLBP = recurrent low back pain; ncCLBP = non-continuous chronic low back pain; cCLBP = continuous chronic low back pain; BMI = body mass index; NRS = numeric rating scale; RMDQ = Roland Morris Disability Questionnaire; RPE = rate of perceived exertion; n = number; m = male; f = female; L = left; R = right; Bi = bilateral)

	HC (*n* = 21)	RLBP (*n* = 23)	ncCLBP (*n* = 15)	cCLBP(*n* = 16)
***Age (years)***	*40* [20–55]	27 [21–53]	31 [20–45]	50 [23–64]
***BMI (kg/m²)***	*23* [18–30]	23 [19,20,21,22,23,24,25,26,27,28,29]	23 [20,21,22,23,24,25,26]	24 [20–32]
***Sex***	*m = 9(43 %), f = 12(57 %)*	*m = 9(39 %), f = 14(61 %)*	*m = 7(47 %), f = 8(53 %)*	*m = 8(50 %), f = 8(50 %)*
***Symptom site***		L = 2(9 %), R = 4(17 %), Bi = 17(74 %)	R = 1(7 %), Bi = 14(93 %)	L = 2(13 %), R = 2(13 %, Bi = 12(74 %)
***Duration of LBP (months)***		112	104	198
***Disability (RMDQ)***	*0* [0–2]	1 [0–6]	5 [1,2,3,4,5,6,7,8,9,10,11]	5 [1,2,3,4,5,6,7,8,9,10,11,12]
***Pain before exercise (NRS)***	*0 [0–0]*	0 [0–1]	1 [0–7]	3 [0–8]
***Pain during exercise (NRS)***	*0* [0–3]	1 [0–6 ]	3 [0–6]	3 [0–8]
***Pain after exercise (NRS)***	*0* [0–2]	1 [0–4]	2 [0–5]	2 [0–8]
***Pain expected pain (NRS)***	*1* [0–4]	2 [0–5]	3 [0–6]	3 [1,2,3,4,5,6,7,8]
***RPE (Borg)***	*9* [6,7,8,9,10,11,12,13,14]	9 [6,7,8,9,10,11,12,13]	11 [7,8,9,10,11,12,13]	11 [6,7,8,9,10,11,12,13,14,15]
***Assisted weight***	*-18* [-26- -15]	-20 [-30 - -13]	-21 [-25 - -13]	-22 [-35 - -15]

### Pain measurements, perceived exertion and disability

Significant differences between groups were found for pain before (*p* < 0.001), during (*p* < 0.001) and after (*p* < 0.001) exercise, as well as for expected pain (*p* < 0.001) (Table 2). No differences between groups were found for rate of perceived exertion.

Before exercise, patients with continuous CLBP and non-continuous CLBP indicated higher pain intensity compared to RLBP (*p* < 0.001; *p* < 0.001 respectively) and healthy controls (*p* < 0.001; *p* < 0.001). No significant differences in pain before exercise was seen between healthy controls and RLBP and between continuous and non-continuous CLBP.

During exercise, lower pain intensities were reported by healthy controls compared to RLBP (*p* = 0.001), non-continuous CLBP (*p* < 0.001) and continuous CLBP (*p* < 0.001). No differences in pain during exercise were seen between the LBP groups.

After exercise, healthy controls reported significant lower pain compared to RLBP (*p* = 0.001), non-continuous (*p* < 0.001) and continuous CLBP (*p* < 0.001). Furthermore, patients with RLBP had significant lower pain afterwards compared to continuous CLBP (*p* < 0.001). No differences were found between non-continuous CLBP and RLBP or between non-continuous CLBP and continuous CLBP.

Pain expected after exercise was significantly higher in non-continuous and continuous CLBP compared to healthy controls (*p* = 0.003; *p* < 0.001) and in continuous CLBP compared to RLBP (*p* = 0.003). No differences were found between healthy controls and RLBP, non-continuous and RLBP and non-continuous and continuous CLBP.

Patients with continuous and non-continuous CLBP were more disabled compared to healthy controls and patients with RLBP (*p* < 0.001). Furthermore, patients with RLBP experienced higher levels of disability compared to healthy controls (*p* < 0.001). No difference in disability was found between patients with continuous and non-continuous CLBP.

Regarding the site of symptoms, no significant differences between groups were observed.

### EMG measurement

A significant interaction effect for phase and group was found for the ES and MF muscles (*p* = 0.001 & *p* = 0.004), but not for LD or GM (*p* > 0.05).

Patients with continuous CLBP showed higher ES and MF EMG activity in the holding phase compared to healthy controls (*p* = 0.015; *p* = 0.013) and RLBP (*p* = 0.035; *p* = 0.037). Also in the concentric phase, significant higher EMG activity was seen in continuous CLBP compared to healthy controls in ES (*p* = 0.011) and in MF (*p* = 0.009). Furthermore, significant higher EMG activity is seen in the concentric phase in the continuous CLBP group compared to the RLBP for the MF muscle (*p* = 0.046), but not for the ES muscle (*p* = 0.062). No differences in ES or MF muscle activity were found between healthy controls and RLBP, between healthy controls and non-continuous CLBP, between RLBP and non-continuous CLBP and between non-continuous CLBP and continuous CLBP. Finally, no differences between groups were found for LD or GM in either the concentric, holding or eccentric phase. Moreover, no differences between groups were found for any muscle in the eccentric phase. Details on the EMG measurements can be found in Table 3.

**Table 3:** Average EMG amplitude during the modified Biering Sorensen’s test, normalized to the MVC reference. All values are expressed by mean difference (%), confidence intervals and p-values (HC = healthy controls; RLBP = recurrent low back pain; ncCLBP = non-continuous chronic low back pain; cCLBP = continuous chronic low back pain; LD = latissimus dorsi muscle; GM = gluteus maximus muscle; ES = upper lumbar erector spinae muscle; MF = multifidus muscle)

		Concentric phase		Holding phase		Eccentric phase	
		**Estimates [CI]**	**p-value**	**Estimates [CI]**	**p-value**	**Estimates [CI]**	**p-value**
**LD**	*∆HC-RLBP*	-0.070 [-1.429;1.290]	1.000	-0.070 [-1.429;1.290]	1.000	-0.064 [-1.424;1.296]	1.000
	*∆HC-ncCLBP*	-1.011 [-2.565;0.544]	0.492	-0.999 [-2.553;0.556]	0.513	-0.828 [-2.383;0.726]	0.916
	*∆HC-cCLBP*	-0.785 [-2.280;0.710]	0.953	-0.832 [-2.327;0.663]	0.813	-0.928 [-2.423;0.567]	0.579
	*∆RLBP-ncCLBP*	-0.941 [-2.468;0.586]	0.594	-0.929 [-2.456;0.598]	0.619	-0.764 [-2.291;0.763]	1.000
	*∆RLBP-cCLBP*	-0.715 [-2.182;0.752]	1.000	-0.762 [-2.229;0.704]	0.977	-0.864 [-2.331;0.603]	0.687
	*∆NcCLBP-cCLBP*	0.226 [-1.422;1.875]	1.000	0.167 [-1.482;1.816]	1.000	-0.100 [-1.748;1.549]	1.000
**GM**	*∆HC-RLBP*	-0.036 [-1.779;1.706]	1.000	-0.034 [-1.777;1.708]	1.000	-0.023 [-1.766;1.719]	1.000
	*∆HC-ncCLBP*	-0.217 [-2.203;1.768]	1.000	-0.318 [-2.303;1.668]	1.000	-0.183 [-2.169;1.802]	1.000
	*∆HC-cCLBP*	-1.240 [-3.149;0.670]	0.497	-1.739 [-3.649;0.170]	0.095	-0.837 [-2.746;1.073]	1.000
	*∆RLBP-ncCLBP*	-0.181 [-2.137;1.775]	1.000	-0.283 [-2.239;1.672]	1.000	-0.160 [-2.116;1.796]	1.000
	*∆RLBP-cCLBP*	-1.203 [-3.082;0.675]	0.522	-1.705 [-3.584;0.173]	0.097	-0.814 [-2.296;1.065]	1.000
	*∆NcCLBP-cCLBP*	-1.022 [-3.128;1.084]	1.000	-1.422 [-3.528;0.684]	0.429	-0.653 [-2.760;1.453]	1.000
**ES**	*∆HC-RLBP*	-0.152 [-0747;0.443]	1.000	-0.085 [-0.680;0.510]	1.000	-0.036 [-0.632;0.559]	1.000
	*∆HC-ncCLBP*	-0.291 [-0.971;0.389]	1.000	-0.325 [-1.005;0.355]	1.000	-0.133 [-0.813;0.547]	1.000
	*∆HC-cCLBP*	-0.774 [-1.425;-0.122]	0.011*	-0.755 [-1.407;-0.103]	0.015*	-0.366 [-1.018;0.286]	0.796
	*∆RLBP-ncCLBP*	-0.139 [-0.809;0.530]	1.000	-0.240 [-0.909;0.429]	1.000	-0.096 [-0.766;0.573]	1.000
	*∆RLBP-cCLBP*	-0.622 [-1.262;0.019]	0.062	-0.670 [-1.310;-0.030]	0.035*	-0.330 [-0.970;0.311]	1.000
	*∆NcCLBP-cCLBP*	-0.482 [-1.202;0.238]	0.442	-0.430 [-1.150;0.290]	0.660	-0.23 [-0.953;0.487]	1.000
**MF**	*∆HC-RLBP*	-0.300 [-1.512;0.913]	1.000	-0.203 [-1.416;1.010]	1.000	-0.116 [-1.329;1.097]	1.000
	*∆HC-ncCLBP*	-0.615 [-2.001;0.771]	1.000	-0.681 [-2.067;0.705]	1.000	-0.198 [-1.584;1.188]	1.000
	*∆HC-cCLBP*	-1.620 [-2.953;-0.287]	0.009*	-1.563 [-2.896;-0.230]	0.013*	-0.809 [2.142;0.525]	0.629
	*∆RLBP-ncCLBP*	-0.315 [-1.675;1.045]	1.000	-0.478 [-1.838;0.882]	1.000	-0.082 [-1.442;1.278]	1.000
	*∆RLBP-cCLBP*	-1.320 [-2.627;-0.014]	0.046*	-1.360 [-2.666;-0.054]	0.037*	-0.693 [-1.999;0.614]	0.932
	*∆NcCLBP-cCLBP*	-1.005 [-2.473;0.463]	0.406	-0.882 [-2.350;0.586]	0.648	-0.611 [-2.079;0.857]	1.000

*statistically significant (*p* < 0.05)

## Discussion

This study intended to establish differences in myoelectrical lumbar muscle activity between healthy subjects and patients with RLBP, continuous and non-continuous CLBP proportionally to the continuation of pain complaints. This study could partly confirm the initial hypothesis. Results show that muscle activity in the ES and MF in the concentric and holding phase was higher in continuous CLBP compared to healthy people. Similarly, enhanced muscle activity was established in the MF and ES in continuous CLBP compared to RLBP, except for ES in the concentric phase (*p* = 0.062). However, no differences between groups were found for GM and LD in any phase. Finally, no differences were detected between non-continuous and continuous CLBP nor between the RLBP patients and healthy persons, at any phase of the exercise.

The enhanced muscle activity of MF and ES in continuous CLBP compared to asymptomatic subjects in both the concentric and the holding phase confirms previous research establishing higher trunk muscle activation in the CLBP population compared with pain-free subjects during functional tasks performance and during extension exercises since movement of upper and lower extremities as well as the upper body threaten the spinal stability. The increased muscle activity may be interpreted as a functional neuromuscular adaptation strategy to improve the reduced spinal stability in people with CLBP [4, 20, 21]. Moreover, based on previous literature, increased muscle activity has also been identified as a response to pain, as a protection for passive subsystem damage, which will in turn increase the pain in a vicious cycle [22].

Furthermore, the enhanced muscle activity between the continuous CLBP and RLBP patients in the ES (in the holding phase) and MF (in the concentric and holding phase) confirms the limited previous research establishing enhanced muscle activity in the lumbar muscles in continuous CLBP compared to RLBP. Goubert et al. (2017) [12] evaluated the metabolic muscle activity during trunk extension and the amount of fat infiltration in the lumbar MF and ES by muscle functional magnetic resonance imaging in patients with continuous CLBP, non-continuous CLBP and RLBP. The performed metabolic muscle activity and amount of muscle fat infiltration was higher in continuous CLBP compared with RLBP. This study also found a positive correlation between muscle activity and the amount of fat infiltration. A higher amount of fat tissue in the lumbar muscles entails a higher workload for the remaining muscles fibers inducing inefficient muscle work, and might therefore explain the enhanced muscle activity found in this study [12]. Therefore, according to this previous research, the increased trunk muscle activity in the continuous CLBP group compared to the RLBP patients found in our study could be a compensatory strategy for the limited lumbar muscle efficiency due to fat infiltration.

No significant differences between groups could be found in the eccentric phase. Previous research established overall muscle activity in the concentric phase is higher compared to the eccentric phase [15, 23, 24], possibly due to a decreased muscle activation in the eccentric phase [22]. Since differences in muscle activity between groups might be more subtle in the eccentric phase and the non-significant differences point in the same direction, the power of the study sample might be too small to detect any significant changes between groups in the eccentric phase.

No differences between the LBP groups were found for the GM or LD muscle activity. This is in accordance with a previous study establishing only limited activation of the GM and LD during the modified Biering-Sorenson exercise [15].

We could not find evidence for alterations in RLBP in remission compared to healthy controls. This is however in contrast with findings of previous research showing either lower muscle activity in the MF during pain-free episodes in RLBP [9], or enhanced paraspinal activity [10, 11] and increased metabolic activity [25] compared to healthy controls. It has been suggested that these altered patterns are a beneficial adaptive short-term strategy to enhance the reduced spinal stability in individuals with RLBP and to initially protect the spine. However, in the long term, this altered muscle activity augments load on spinal structures, which may enhance the risk of reinjury and predispose to recurrent LBP episodes [9, 26], or even the transition to CLBP. However, the mean age of the RLBP in the current study is low. It is possible that young RLBP patients in pain remission are able to restore muscle activity between pain flares, which results in a muscle activity (more) comparable to healthy persons, leading to a lack of significant differences between both groups. However further research is needed to confirm this hypothesis.

No differences in lumbar muscle activity were found between the non-continuous CLBP and the RLBP or the continuous CLBP. Taken together with the above-mentioned results, this study might suggest that the LBP population represents itself as a spectrum, in which muscle activity in MF and ES in the holding and concentric phase is normal in healthy persons and increases gradually with the amount of pain days in LBP populations. These findings provide a potential pathophysiological mechanism for the increased likelihood of recurrence and maintenance of the LBP condition. The pain intensity before, during and after exercise, disability and rate of perceived exertion in this LBP population are also positioned along a continuum being the lowest in RLBP, increasing in non-continuous CLBP and the highest in continuous CLBP. Although the distributions represent a spectrum of LBP, it should be highlighted that a large heterogeneity exists in the individual pain and disability scores, indicating treatment strategies should always be individualized.

In our study, both CLBP groups indicated higher expected pain due to exercise compared to asymptomatic participants and RLBP, although only a borderline higher expected pain was noted in the non-continuous CLBP group compared to the RLBP group. These results are in line with previous studies establishing the relationship between expectancy of pain and pain-related fear for movement [27]. Furthermore, also hypervigilance is correlated to higher amounts of pain [28]. It is therefore possible that the enhanced muscle activity measured in the CLBP groups is a maladaptive protection strategy of patients due to fear of pain following the exercise.

From a clinical point of view, this study offers additional knowledge that muscle activity is higher in patients with continuous CLBP compared to RLBP, and non-continuous CLBP seem to float in between. These findings highlight the importance of strategies in normalizing muscle activity in the three LBP groups during rehabilitation. However, there are some limitations that must be considered. The amount of physical activity of the participants was not registered in the current study. Since the positive influence of an active lifestyle on spinal control has already been demonstrated [29], participants with higher levels of physical activity might present a lower activation of the lumbar muscles and therefore a more favorable spinal motion control. Moreover, the used surface EMG was not able to specify muscle activity between superficial and deep fibers of the MF. Furthermore, electromyographic activity of the ES and MF was measured at levels L1 and L5 respectively. Thus, the findings of the current study cannot be extrapolated to other low back levels. Finally, since many comparisons were made in this study, a type I error cannot be ruled out.

## Conclusions

In summary, continuous CLBP patients present increased ES and MF muscle activity during the holding and concentric phase of an extension exercise compared to healthy controls and to a lesser extent compared to RLBP patients. No differences in muscle activity are found between RLBP and healthy persons and between non-continuous CLBP and all other groups. No differences in muscle activity are found for GM and LD.

## Data Availability

The datasets used and/or analysed during the current study are available from the corresponding author on reasonable request.

## Abbreviations

LBP: Low back pain
EMG: electromyographic
ES: erector spinae
MF: multifidus
LD: latissimus dorsi
GM: gluteus maximus
CLBP: chronic low back pain
RLBP: recurrent low back pain
1RM: one repetitions maximum
MVC: maximal voluntary contraction
RPE: rate of perceived exertion
