# Correction to: Differences in myoelectric activity of the lumbar muscles between recurrent and chronic low back pain: a cross-sectional study

**DOI:** 10.1186/s12891-021-04732-5

**Published:** 2021-10-22

**Authors:** Mercè Balasch-Bernat, Tine Willems, Lieven Danneels, Mira Meeus, Dorien Goubert

**Affiliations:** 1 Department of Physiotherapy, University of Valencia, Valencia, Spain; 2 Physiotherapy in Motion, MultiSpeciality Research Group (PTinMOTION), University of Valencia, Valencia, Spain; 3 Department of Rehabilitation Sciences, Faculty of Medicine and Health Sciences, Ghent University, Ghent, Belgium; 4 Department of Rehabilitation Sciences, Campus Heymans (UZ) 3 B3, Corneel Heymanslaan 10, 9000 Ghent, Belgium; 5 Pain in Motion Research Group, Valencia, Spain; 6 Department of Rehabilitation Sciences and Physiotherapy, Faculty of Medicine and Health Sciences, University of Antwerp, Antwerp, Belgium

**Correction to: BMC Musculoskelet Disord 22, 756 (2021)**

**https://doi.org/10.1186/s12891-021-04623-9**

Following the publication of the original article [1] the authors noticed that the reference for the manuscript is incorrect; the authors’ first and last names were swapped.

The reference should be:

Balasch-Bernat, M., Willems, T., Danneels, L., Meeus M. and Goubert, D. Differences in myoelectric activity of the lumbar muscles between recurrent and chronic low back pain: a cross-sectional study. *BMC Musculoskelet Disord* 22, 756 (2021). 10.1186/s12891-021-04623-9.

The original article [1] has been updated.
